# Supplementary material for: FERN – a Java framework for stochastic simulation and evaluation of reaction networks
Source: BMC Bioinformatics. 2008 Aug 29;9:356. doi: 10.1186/1471-2105-9-356 (PMC2553347; doi:10.1186/1471-2105-9-356)
Supplement: Additional file 1 — FERN distribution, Version 1.3. This archive contains the FERN source code and binaries as well as documentation and example models in FernML and SBML. [file 1471-2105-9-356-S1.zip › fern/doc/javadoc/index-files/index-14.html]

O-Index


---


|  |  |  |
| --- | --- | --- |
| **Overview** \| Package \| Class \| Use \| **Tree** \| **Deprecated** \| **Index** \| **Help** | |  |
| **PREV LETTER**   **NEXT LETTER** | **FRAMES**    **NO FRAMES**     **All Classes** |  |


A B C D E F G H I J L M N O P R S T U V 

---


## **O**

**Observer** - Class in fern.simulation.observer: Implementing classes are used to observe certain aspects of simulations. **Observer(Simulator)** - Constructor for class fern.simulation.observer.Observer: Creates an observer dedicated to one simulator. **OrController** - Class in fern.simulation.controller: Implements a `SimulationController` composed of many `SimulationController`. **OrController(SimulationController...)** - Constructor for class fern.simulation.controller.OrController: **originalNetwork** - Variable in class fern.analysis.AnalysisBase: Contains the original network if `network` is a `ModifierNetwork`. **OverviewPane** - Class in fern.cellDesigner.ui: **OverviewPane(MainFrame)** - Constructor for class fern.cellDesigner.ui.OverviewPane: **OverviewPane** - Class in fern.cytoscape.ui: **OverviewPane(MainFrame)** - Constructor for class fern.cytoscape.ui.OverviewPane

---


|  |  |  |
| --- | --- | --- |
| **Overview** \| Package \| Class \| Use \| **Tree** \| **Deprecated** \| **Index** \| **Help** | |  |
| **PREV LETTER**   **NEXT LETTER** | **FRAMES**    **NO FRAMES**     **All Classes** |  |


A B C D E F G H I J L M N O P R S T U V 

---
